# On Guarana

**Published:** 1852-09

**Authors:** D. Ritchie


### MATERIA MEDICA AND THERAPEUTICS.

*On Guaraná.* By D. RITCHIE, Esq.—Guaraná is a substance used in Brazil as a medicine. It is prepared from the seeds of the *paullinia sorbilis*, a sapindaceous plant. The masses of which it consists are cylindrical or spherical, hard, uneven, brown or black on the surface; fracture conchoidal, unequal, resinoid; color reddish brown, like chocolate. It is liable to be adulterated with cocoa or mandiocca flour. Chemical analysis shows the presence of tannin, caffeine and guaranine.

The medicinal properties have been examined by M. Theodore Martius, and more particularly by Dr. Gavrelle, who was physician to Don Pedro in Brazil. By the vulgar it is held to be stomachic, antifebrile and aphrodisiac; it is used in dysentery, diarrhœa, retention of urine and various other affections. It stimulates, and at the same time soothes the gastric plexus of nerves. It reduces the excited sensibility of the cæliac plexus, thereby diminishing febrile action, and strengthening the stomach and intestines, particularly restraining excessive mucous discharges, increasing the action of the heart and arteries, and promoting diaphoresis. It is therefore indicated in fever, or reduced vital power from cold or prolonged wet, grief, too great muscular exertion, depression of spirits, long watching, and also in colic, flatulence, anorexia, nervous hemicrania, or in a dry condition of the skin. It is contra-indicated in a plethoric or loaded condition of the abdominal viscera, and in determination of blood to the head. It is said to increase the venereal appetite, but to diminish the fecundating power. It is a valuable remedy in irritation of the urethra and bladder, succeeding venereal or attending organic disease.

Guaraná, in the state of powder, is given in doses of ʒj. three or four times daily, with water and sugar, or with syrup and mucilage, conjoined with an aromatic. A convenient form is the extract, made by treating guaraná with alcohol; it may be exhibited in the form of solution or pills.—*London Journal of Medicine.*

